# Supplementary figures and images for: Immune signatures of SARS-CoV-2 infection resolution in human lung tissues
Source: PLoS Pathog. 2025 Sep 8;21(9):e1013469. doi: 10.1371/journal.ppat.1013469 (PMC12425302; doi:10.1371/journal.ppat.1013469)

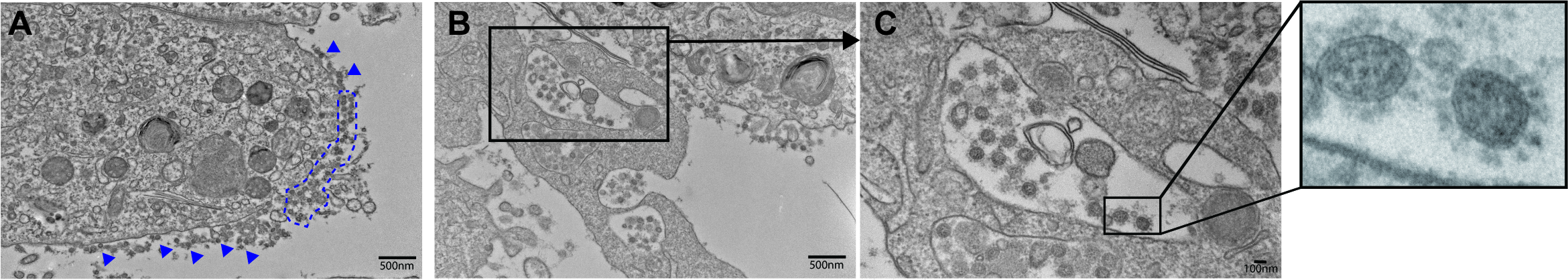

Supplement: S1 Fig — (A-C) Transmission electron microscopy (TEM) of fLX tissue sections extracted from BLT-L mice at 2 dpi, illustrating virus particles at the cell surface as indicated by the blue arrows and blue dotted line (A) and viral particles in AT2 cells as evident by the presence of lamellar bodies (B,C). Scale bars are indicated in the images. (TIF) [file ppat.1013469.s001.tif]

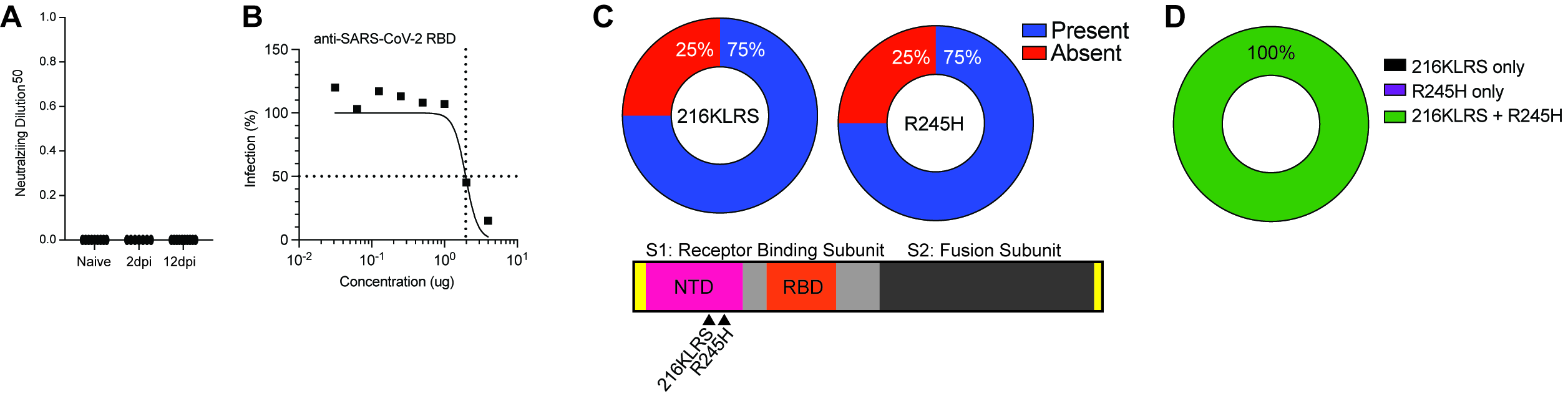

Supplement: S2 Fig — (A) Neutralizing efficacy (ND50 values) of serum extracted from naïve or infected BLT-L mice (2 and 12 dpi). Assay was performed using VeroE6 cells and a recombinant WA-1 SARS-CoV-2 virus expressing NanoLuc. (B) Titration of the neutralizing activity of an anti-RBD antibody (serving as positive control) against WA-1 SARS-CoV-2 virus expressing NanoLuc. (C-D) SARS-CoV-2 virus isolated from 2 dpi fLX was deep sequenced and assessed for mutations relative to the inoculation (WA-1) strain. C Upper panel: Pie chart depicting the percentage of fLX with virus containing 216KLRS insertion and R245H non-synonymous mutation. C Lower Panel: Schematic representation of SARS-CoV-2 spike protein highlighting the location of mutations. Not to scale. D: Pie chart depicting the percentage of fLX with virus that showed signs of co-evolution of 216KLRS and R245H. (TIF) [file ppat.1013469.s002.tif]

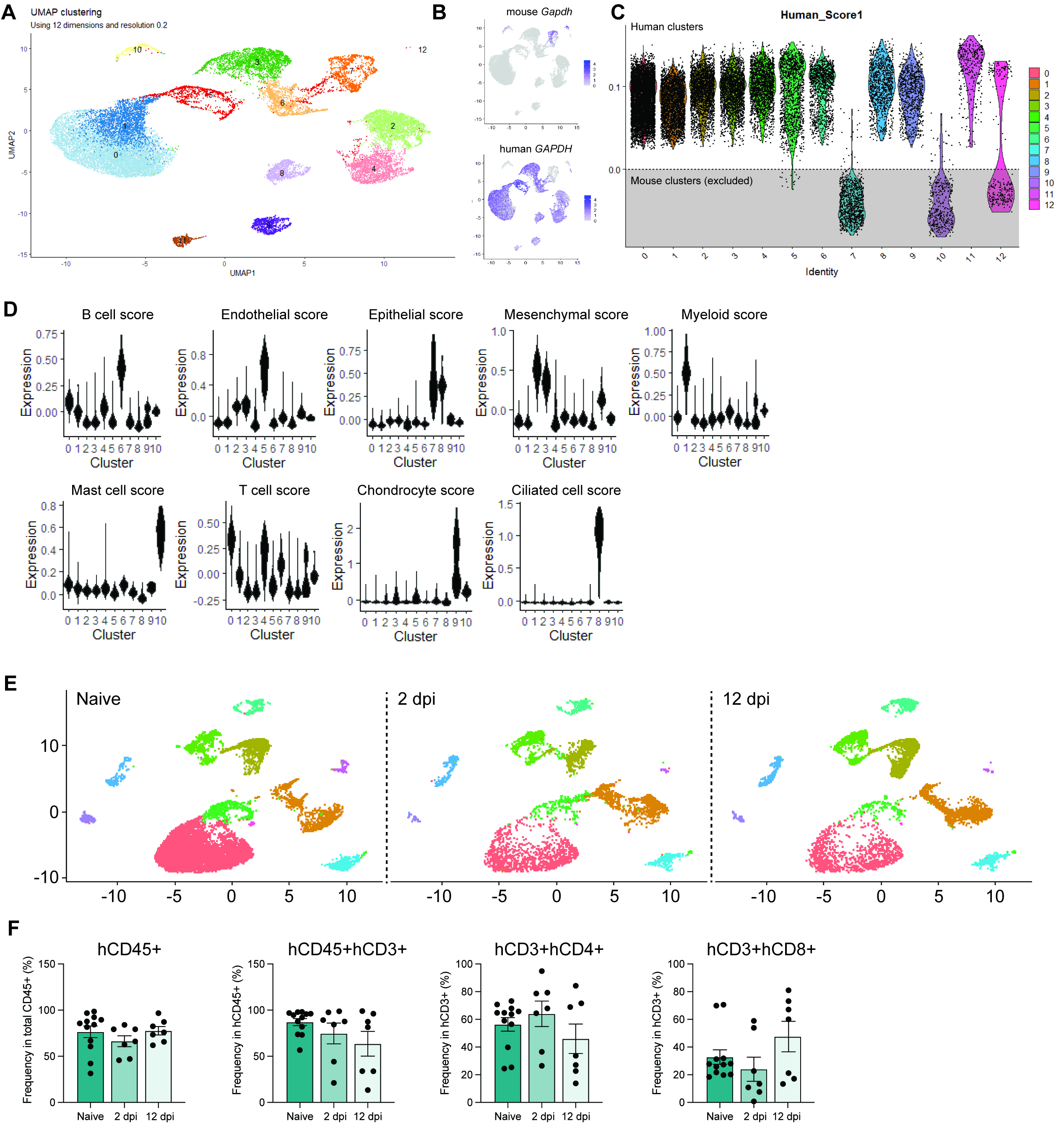

Supplement: S3 Fig — (A-E) Single-cell RNA sequencing was performed on naïve fLX and infected fLX (at 2- and 12 dpi). Sequencing reads were aligned to a combined human, mouse and SARS-CoV-2 viral genome. (A) UMAP plot clustering on all cell (human and mouse) populations detected. (B) Expression of mouse (top) and human GAPDH (bottom) in all clusters. (C) A human score was applied to each cluster to identify human clusters (above dotted line) and mouse clusters (below dotted line; gray zone). Mouse clusters were removed from downstream analysis. (D) Cell scoring system applied to human cell clusters to classify each cluster. (E) UMAP clustering of human cells separated by time point (naïve: left, 2 dpi: center, and 12 dpi: right). (F) Flow cytometric analysis showing frequency of hCD45 + , hCD3 + , hCD4 + , and hCD8 + cells among PBMCs extracted from the blood of naïve and infected BLT-L mice at 2-, 6-, and 12 dpi. (TIF) [file ppat.1013469.s003.tif]

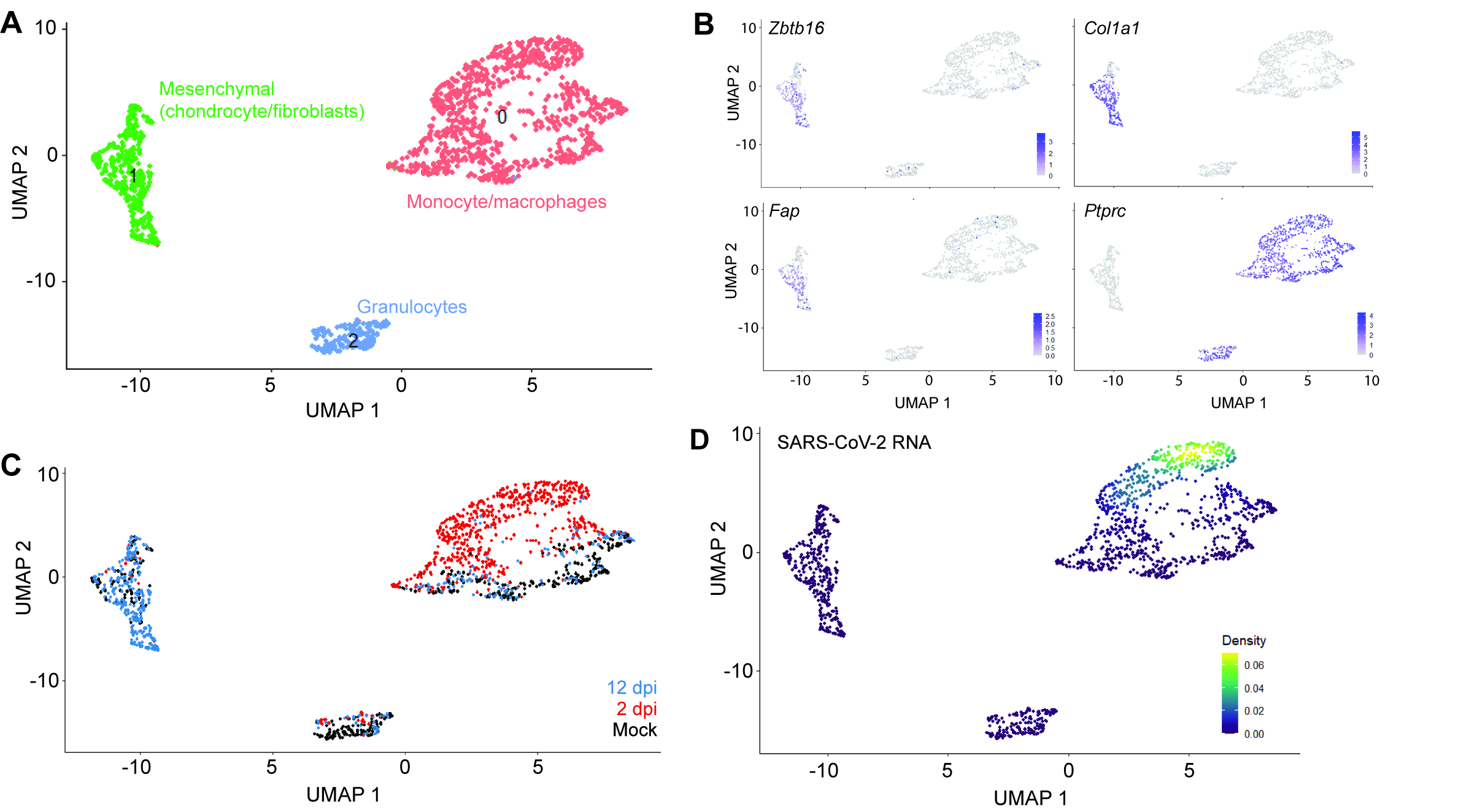

Supplement: S4 Fig — Single-cell RNA sequencing was performed on naïve fLX and infected fLX (at 2- and 12 dpi). Sequencing reads were aligned to a combined human, mouse and SARS-CoV-2 viral genome. (A) UMAP plot clustering of the mouse cell compartment of naïve fLX and fLX at 2 and 12 dpi. (B) UMAP plot showing expression of select genes used for annotating mouse clusters. (C) Temporal annotation of the mouse clusters on the UMAP plot: naïve (black), 2 dpi (red), 12 dpi (blue). (D) UMAP plot showing the distribution of SARS-CoV-2 viral RNA transcripts by density across all mouse cell clusters and time points analyzed by scRNA-seq. (TIF) [file ppat.1013469.s004.tif]

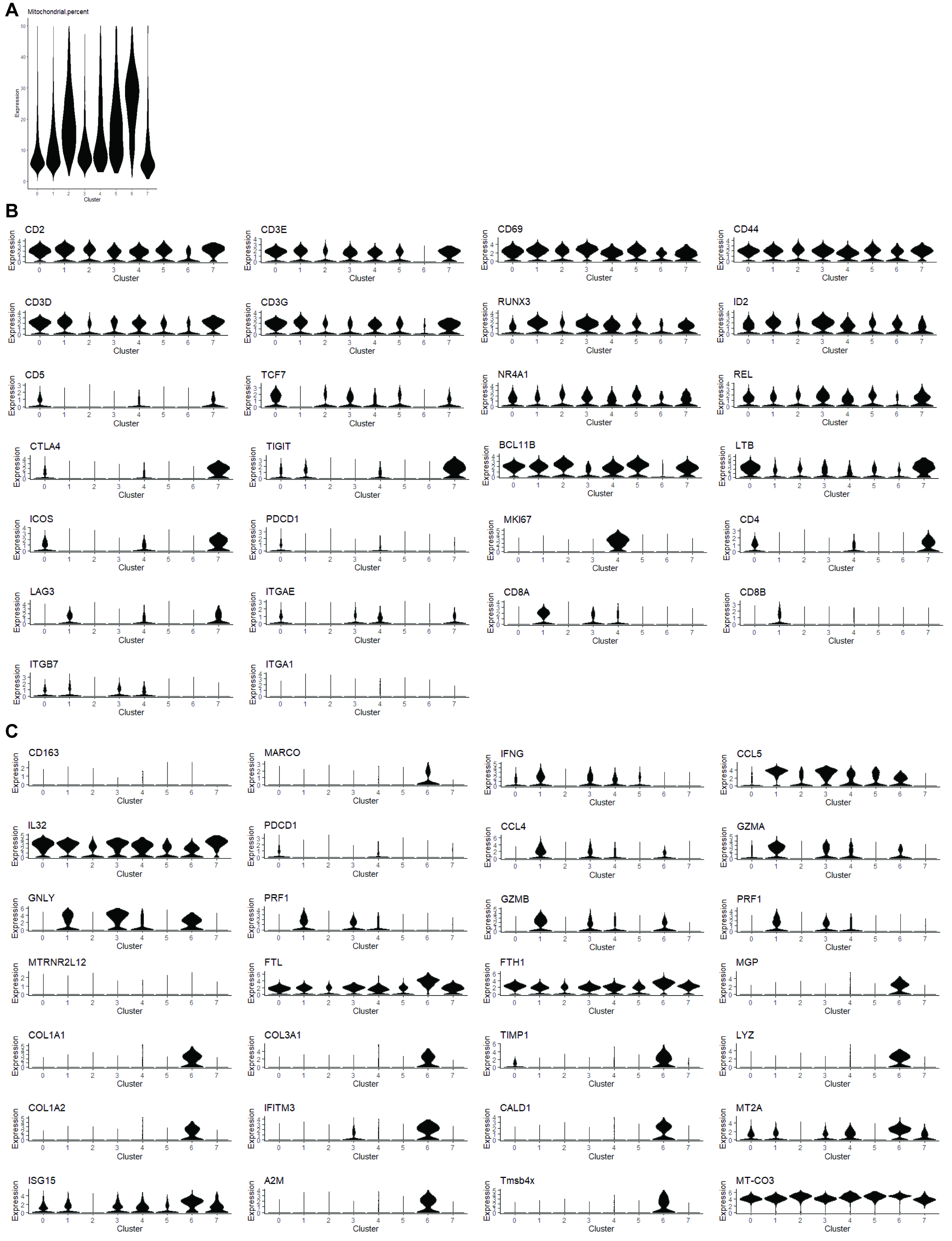

Supplement: S5 Fig — (A) Mitochondrial gene expression among the different T cell sub-clusters. (B) Expression of T-cell-associated genes among the different T-cell sub-clusters. (C) Expression of myeloid/macrophage-associated genes among the different T-cell sub-clusters. (TIF) [file ppat.1013469.s005.tif]

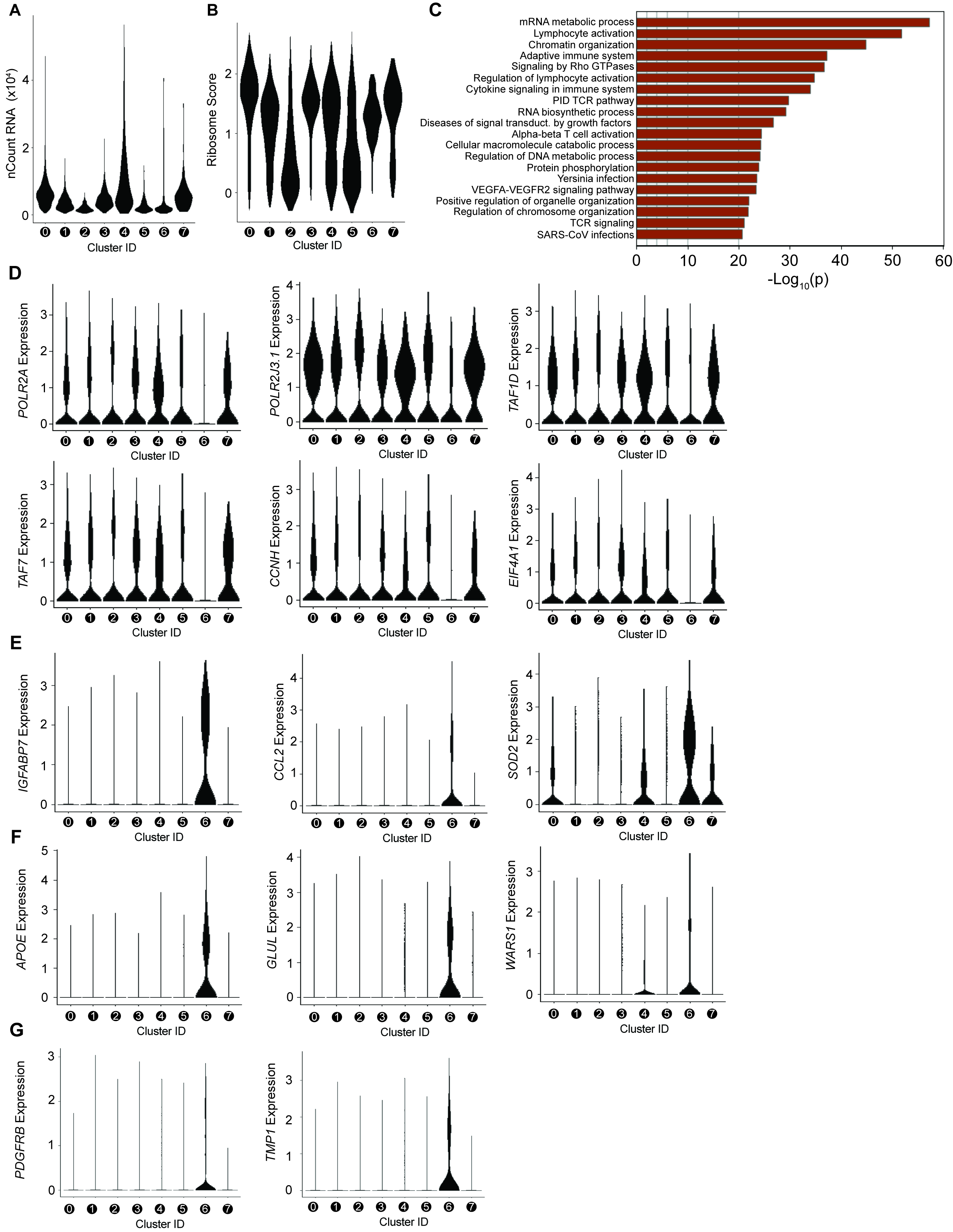

Supplement: S6 Fig — (A) Number of total RNA counts in T cell clusters. (B) Ribosomal score for each T cell cluster. (C) Pathway analysis of significantly downregulated genes in macrophage-like cell cluster (cluster 6). (D) Expression of select genes related to mRNA translation across T cell clusters. (E) Expression of select genes related to cellular stress responses across T cell clusters. (F) Expression of select genes related to macromolecule biosynthesis in T cell clusters. (G) Expression of select genes related to non-membrane-bounded organelle assembly. (TIF) [file ppat.1013469.s006.tif]

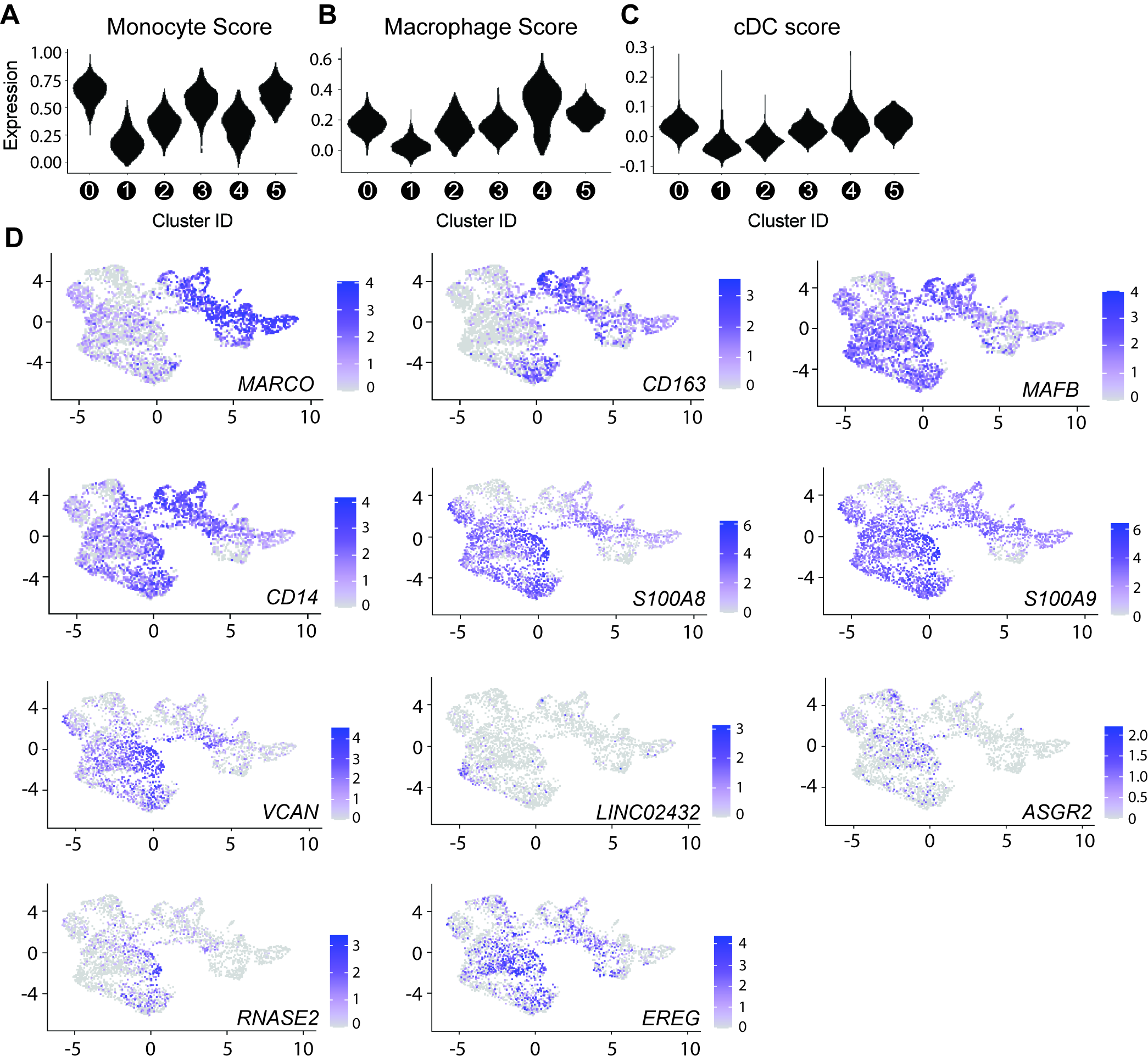

Supplement: S7 Fig — (A-C) Violin plot displaying monocyte (A), macrophage (B) and dendritic cell (cDC) (C) gene signature score for each myeloid sub-cluster. (D) UMAP plots representing the expression of several myeloid markers that were employed to further define the identity of the different myeloid sub-clusters. (TIF) [file ppat.1013469.s007.tif]

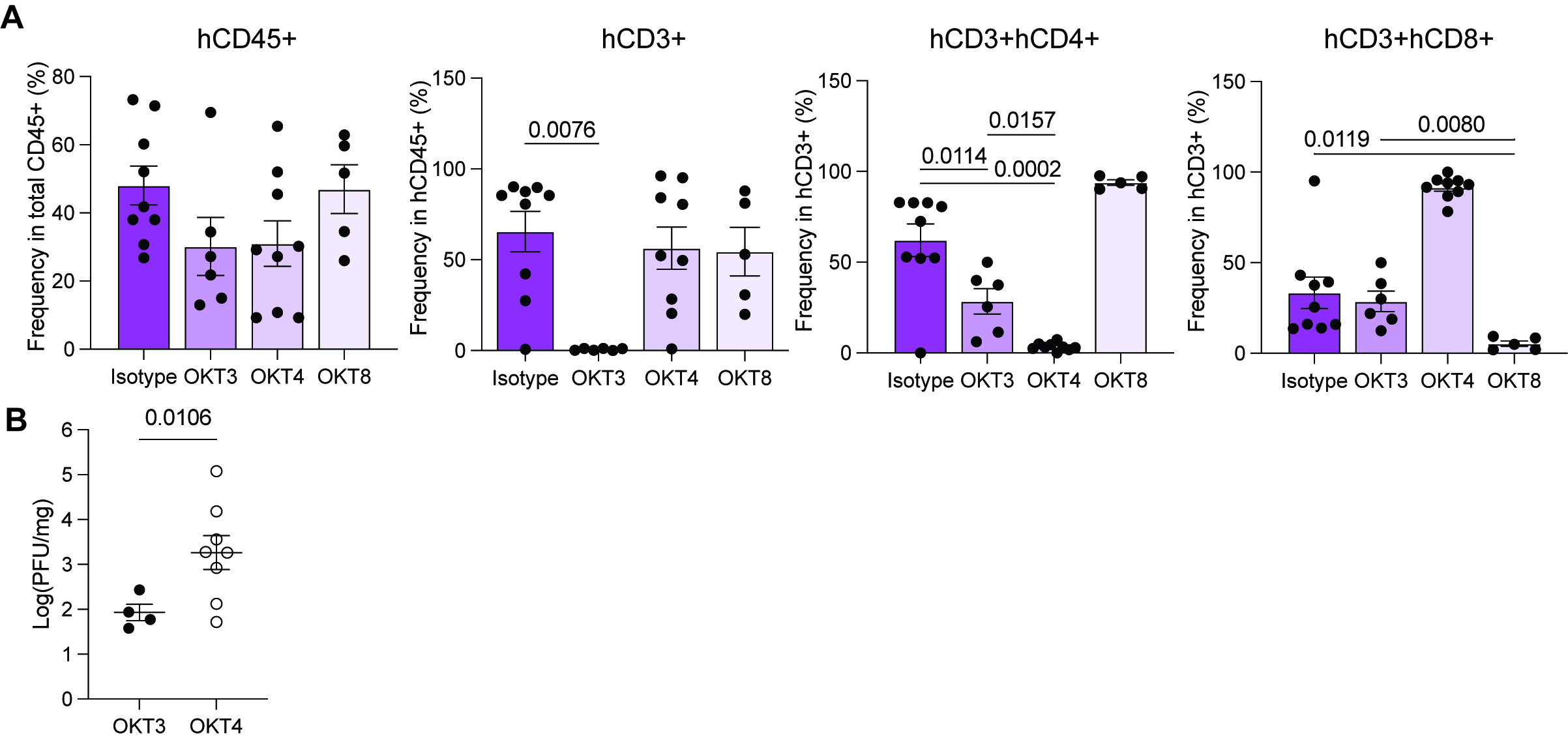

Supplement: S8 Fig — (A) Flow cytometric analysis showing the frequency of hCD45 + , hCD3 + , hCD3 + hCD4 + , and hCD3 + hCD8 + cells among PBMCs extracted from the blood of BLT-L mice treated with an isotype antibody, OKT3, OKT4 or OKT8 antibody. Error bars represent mean ± SEM. One-way ANOVA. P-values are indicated on the graphs. (B) Viral titer (log(PFU/mg)) of CD3 + cell (OKT3) and CD4 + cell-depleted (OKT4) fLX that were positive for viral infection. Unpaired, non-parametric T-test. P-value is indicated on the graph. (TIF) [file ppat.1013469.s008.tif]
