# Supplementary material for: Immune signatures of SARS-CoV-2 infection resolution in human lung tissues
Source: PLoS Pathog. 2025 Sep 8;21(9):e1013469. doi: 10.1371/journal.ppat.1013469 (PMC12425302; doi:10.1371/journal.ppat.1013469)
Supplement: S1 Table — Detailed histopathological scoring for each fLX analyzed in this study. (DOCX) [file ppat.1013469.s009.docx]

| **Condition** | **Syncytial cells** | **Hyaline Membrane** | **Neutrophils in Airspace** | **Airspace**  **Hemorrhage** | **Interstitial and/or airspace edema** | **Desquamated pneumocytes** | **Intra-airspace necrosis** | **Capillary fibrin thrombi** | **Intermediate to large vessel fibrin thrombi** | **Coagulative necrosis affiliated with fibrin thrombi** | **Cumulative Score** |
| --- | --- | --- | --- | --- | --- | --- | --- | --- | --- | --- | --- |
| 12 dpi | 0 | 0 | 0 | 0 | 1 | 0 | 0 | 0 | 0 | 0 | 1 |
| 12 dpi | 0 | 0 | 0 | 0 | 1 | 0 | 0 | 1 | 0 | 0 | 2 |
| 12 dpi | 0 | 0 | 0 | 0 | 1 | 1 | 0 | 1 | 2 | 0 | 5 |
| 12 dpi | 0 | 0 | 0 | 0 | 1 | 2 | 1 | 1 | 2 | 0 | 7 |
| 12 dpi | 0 | 0 | 0 | 0 | 1 | 1 | 0 | 2 | 1 | 0 | 5 |
| 12 dpi | 0 | 0 | 0 | 0 | 0 | 0 | 0 | 0 | 0 | 0 | 0 |
| 12 dpi | 0 | 0 | 0 | 0 | 1 | 1 | 0 | 0 | 0 | 0 | 2 |
| 2 dpi | 1 | 0 | 1 | 1 | 2 | 2 | 1 | 2 | 1 | 1 | 12 |
| 2 dpi | 0 | 0 | 2 | 1 | 2 | 2 | 2 | 2 | 3 | 1 | 15 |
| 2 dpi | 0 | 0 | 0 | 0 | 1 | 1 | 0 | 3 | 3 | 0 | 8 |
| 2 dpi | 0 | 0 | 1 | 2 | 2 | 3 | 1 | 1 | 2 | 0 | 12 |
| 2 dpi | 0 | 0 | 3 | 2 | 3 | 2 | 3 | 2 | 3 | 0 | 18 |
| 2 dpi | 0 | 0 | 1 | 0 | 0 | 1 | 1 | 2 | 1 | 0 | 6 |
| 2 dpi | 0 | 0 | 0 | 2 | 1 | 1 | 0 | 2 | 2 | 1 | 9 |
| 2 dpi | 0 | 0 | 2 | 0 | 1 | 1 | 1 | 3 | 3 | 0 | 11 |
| 2 dpi | 0 | 0 | 0 | 0 | 1 | 0 | 0 | 2 | 3 | 0 | 6 |
| 2 dpi | 0 | 0 | 1 | 2 | 1 | 1 | 1 | 2 | 2 | 0 | 10 |
| 6 dpi | 0 | 0 | 0 | 0 | 1 | 0 | 0 | 1 | 1 | 0 | 3 |
| 6 dpi | 0 | 0 | 0 | 1 | 1 | 0 | 0 | 0 | 2 | 0 | 4 |
| 6 dpi | 0 | 0 | 0 | 0 | 2 | 0 | 0 | 0 | 2 | 0 | 4 |
| 6 dpi | 0 | 0 | 0 | 0 | 1 | 0 | 0 | 0 | 1 | 0 | 2 |
| 6 dpi | 0 | 0 | 0 | 0 | 2 | 0 | 0 | 0 | 1 | 0 | 3 |
| 6 dpi | 0 | 0 | 0 | 0 | 1 | 1 | 0 | 0 | 0 | 0 | 2 |
| Naive | 0 | 0 | 0 | 1 | 0 | 0 | 0 | 1 | 2 | 0 | 4 |
| Naive | 0 | 0 | 1 | 0 | 1 | 1 | 1 | 2 | 2 | 0 | 8 |
| Naive | 0 | 0 | 0 | 0 | 1 | 1 | 0 | 1 | 3 | 0 | 6 |
| Naive | 0 | 0 | 0 | 0 | 1 | 0 | 1 | 2 | 1 | 0 | 5 |
| Naive | 0 | 0 | 0 | 0 | 1 | 0 | 0 | 1 | 1 | 0 | 3 |
| Naïve | 0 | 0 | 0 | 0 | 1 | 1 | 0 | 1 | 2 | 0 | 5 |
| Naïve | 0 | 0 | 0 | 0 | 2 | 1 | 0 | 0 | 2 | 0 | 5 |
| Naive | 0 | 0 | 0 | 2 | 1 | 1 | 0 | 1 | 2 | 0 | 7 |
| 12 dpi Isoty. | 0 | 0 | 0 | 0 | 0 | 1 | 0 | 0 | 0 | 0 | 1 |
| 12 dpi Isoty. | 0 | 0 | 0 | 0 | 1 | 1 | 0 | 1 | 1 | 0 | 4 |
| 12 dpi Isoty. | 0 | 0 | 0 | 0 | 1 | 1 | 0 | 0 | 0 | 0 | 2 |
| 12 dpi Isoty. | 0 | 0 | 0 | 0 | 2 | 0 | 0 | 2 | 3 | 0 | 7 |
| 12 dpi Isoty. | 0 | 0 | 0 | 0 | 1 | 0 | 0 | 0 | 0 | 0 | 1 |
| 12 dpi Isoty. | 0 | 0 | 0 | 0 | 2 | 0 | 0 | 1 | 2 | 0 | 5 |
| 12 dpi OKT3 | 0 | 0 | 0 | 0 | 1 | 1 | 0 | 0 | 0 | 0 | 2 |
| 12 dpi OKT3 | 0 | 0 | 0 | 1 | 2 | 0 | 0 | 1 | 2 | 0 | 6 |
| 12 dpi OKT3 | 0 | 0 | 0 | 0 | 2 | 0 | 0 | 0 | 1 | 0 | 3 |
| 12 dpi OKT3 | 0 | 0 | 0 | 0 | 2 | 0 | 0 | 1 | 2 | 0 | 5 |
| 12 dpi OKT4 | 0 | 0 | 0 | 0 | 1 | 1 | 0 | 0 | 2 | 0 | 4 |
| 12 dpi OKT4 | 0 | 0 | 0 | 0 | 2 | 1 | 0 | 0 | 2 | 0 | 5 |
| 12 dpi OKT4 | 0 | 0 | 2 | 0 | 1 | 2 | 1 | 1 | 2 | 0 | 9 |
| 12 dpi OKT4 | 0 | 0 | 2 | 0 | 1 | 1 | 2 | 1 | 2 | 1 | 10 |
| 12 dpi OKT4 | 0 | 0 | 1 | 0 | 1 | 0 | 0 | 0 | 0 | 0 | 2 |
| 12 dpi OKT4 | 0 | 0 | 0 | 0 | 2 | 0 | 0 | 0 | 0 | 0 | 2 |
| 12 dpi OKT4 | 0 | 0 | 0 | 0 | 2 | 0 | 0 | 0 | 2 | 0 | 4 |
| 12 dpi OKT8 | 0 | 0 | 1 | 0 | 1 | 1 | 1 | 1 | 2 | 0 | 7 |
| 12 dpi OKT8 | 1 | 0 | 1 | 0 | 1 | 2 | 1 | 1 | 2 | 0 | 9 |
| 12 dpi OKT8 | 0 | 0 | 1 | 1 | 0 | 1 | 1 | 1 | 2 | 0 | 7 |
| 12 dpi OKT8 | 0 | 0 | 1 | 1 | 1 | 1 | 1 | 0 | 0 | 0 | 5 |
| 12 dpi OKT8 | 0 | 0 | 0 | 0 | 2 | 0 | 0 | 0 | 0 | 0 | 2 |
| 12 dpi OKT8 | 0 | 0 | 0 | 0 | 2 | 0 | 0 | 0 | 0 | 0 | 2 |

**S1 Table. Histopathological scoring of fLX.** Detailed histopathological scoring for each fLX analyzed in this study.
