# Supplementary material for: Immune signatures of SARS-CoV-2 infection resolution in human lung tissues
Source: PLoS Pathog. 2025 Sep 8;21(9):e1013469. doi: 10.1371/journal.ppat.1013469 (PMC12425302; doi:10.1371/journal.ppat.1013469)
Supplement: S2 Table — SARS-CoV-2 mutations identified among viral reads isolated from fLX at 2 dpi and from our concentrated viral stock (i.e., WA1 positive stock used to inoculate fLX), as compared to the Wuhan-1 consensus sequence. (DOCX) [file ppat.1013469.s010.docx]

| Sample | Mutations | Notes |
| --- | --- | --- |
| 2 dpi - 1 | **Spike 216KLRS insertion 99%** | There is a deletion at spike 221, but there is very low coverage (only 2x) |
|  | **Spike R245H - 100%** |  |
| 2 dpi - 2 | orf1ab K1051 insertion - 28% | No mutations in spike, but spike had no coverage at AA sites 216 and 245 |
| 2 dpi - 3 | **Spike 216KLRS insertion 100%** |  |
|  | **Spike R245H - 100%** |  |
| 2 dpi - 4 | **Spike 216KLRS insertion 91%** |  |
|  | **Spike R245H - 100%** |  |
| 2 dpi - 5 | **Spike 216KLRS insertion 81%** |  |
|  | **Spike R245H - 100%** |  |
| 2 dpi - 6 | **Spike 216KLRS insertion 96%** |  |
|  | **Spike R245H - 99%** |  |
|  | NS6 S43P - 30% |  |
| 2 dpi - 7 | **Spike 216KLRS insertion - 98%** |  |
|  | **Spike R245H - 100%** |  |
| 2 dpi - 8 | orf1ab V1866 del (frameshift) - 35% | Low coverage at AA sites 216 and 245, but there is 1 read supporting the presence of both mutations |
|  | orf1ab V3048L - 33% |  |
|  | NS3 - F168 del (frameshift) - 47%, |  |
| WA1 positive stock | Spike E96A - 51% |  |
|  | Spike 678-690 deletion - 87% |  |
|  | NS7b 43 - NS8 1 deletion - 50% |  |

**S2 Table. SARS-CoV-2 mutations in infected fLX.** SARS-CoV-2 mutations identified among viral reads isolated from fLX at 2 dpi and from our concentrated viral stock (i.e., WA1 positive stock used to inoculate fLX), as compared to the Wuhan-1 consensus sequence.
