# Supplementary material for: Immune signatures of SARS-CoV-2 infection resolution in human lung tissues
Source: PLoS Pathog. 2025 Sep 8;21(9):e1013469. doi: 10.1371/journal.ppat.1013469 (PMC12425302; doi:10.1371/journal.ppat.1013469)
Supplement: Attachment — Submitted filename: Kenney et al_Response to Reviewers.docx [file ppat.1013469.s012.docx]

**RESPONSES TO REVIEWERS**

***Part I - Summary*** *Please use this section to discuss strengths/weaknesses of study, novelty/significance, general execution and scholarship.*

*Reviewer #1: The manuscript “Immune Signatures of SARS-CoV-2 Infection Resolution in Human Lung Tissues” by Kenney et al. provides a very comprehensive characterization of immune signatures, using single cell transcriptomics, of resolution of SARS-CoV-2 infection in a human lung xenograft mouse model. The work is a follow-up of the observation by the same group that reconstitution of the human immune system resulted in a transient SARS-CoV-2 infection, rather than prolonged infection observed in lung-only models. The authors identify several cell populations that play a role in this resolution, and provide experimental data supporting the role of CD4+ cells. Overall, the experiments are well designed and the data support the conclusions. However some clarification is needed for interpretation.*

*Reviewer #2: In this manuscript entitled “Immune Signatures of SARS-CoV-2 Infection Resolution in Human Lung Tissues” by Kenney et al. mapped the immunological events during SARS-CoV-2 infection in the lung in a unique mouse model with the mice co-engrafted with a genetically matched human immune system and fetal lung xenografts. In this novel mouse model SARS-CoV-2 infection is rapidly cleared in the fetal lung tissue with rapid recovery of the histopathologic changes seen in SARS-CoV-2 infection. Surprisingly despite having no evidence of a humoral response (due to the limitations of the humanized mouse model system) they still showed evidence of viral evolution/adaptation. Interestingly these histopathologic changes and loss of the AT2 program seen in the lung epithelial cells in human COVID-19 patients was also recapitulated in this model platform. The authors note an alteration and enrichment of a T-cell subcluster that when analyzed more carefully displayed low levels of CD4 and CD8 with high-level expression of macrophage markers which they dubbed double-negative T-cells. Of great interest was the finding that depletion of CD4+ cells led to the development of a prolonged infection with multiple cellular and immune alterations. Moreover, the study shows the important role of infiltrating monocytes in driving resolution of SARS-CoV-2 infection. This is a highly relevant topic, as the delineation of SARS-CoV-2 induced injury and repair is important in understanding lung-pathogen responses and the protective role of the myeloid compartment represents an improved understanding of SARS-CoV-2 infection. Overall, this is manuscript is a robust work with a complex model platform that addresses several open areas in the field however, several issues need to be addressed before the manuscript can be considered for publication*

*Major comments
1. Histopathologic changes seen in the human lung parenchyma are somewhat hard to interpret as no higher power H&E or trichrome images were shown (Fig 2). The histopathologic score which is a unique scoring system developed by the investigators in a prior published manuscript should still be elaborated in this manuscript and in the text as it is not a gold-standard analysis technique and therefore the interpretation of the data is not clear as it is not clear what it being quantified (Fig.2H). It would also add value (if shown in a table in the supplement) what is being quantified and what components of the score system is driving the values seen. Do the investigators have any comment on the differences seen in the score seen in their prior Cell Reports paper and the values seen in this report?*

We thank the reviewer for their insightful comments and appreciate the opportunity to clarify our histopathologic scoring system. To address the request for greater detail, we have added a supplemental table (Table S2) that provides a comprehensive breakdown of the individual fLX samples analyzed, including the specific histopathologic features quantified.

Our histopathologic scoring system was developed in our prior publication and, as noted, is not a gold-standard technique but rather a tailored approach to capture the unique pathology observed in the fLX model. This scoring system focuses on features associated with acute lung injury (ALI), such as necrosis, neutrophil infiltration, hemorrhage, edema, and thrombosis. These features are notably more prominent in fLX engraftments lacking co-engraftment with human hematopoietic cells. In contrast, naïve fLX engraftments occasionally exhibit some of these findings, albeit more subtly and sporadically, which reflects the inherent background pathology of the model.

In the current study, we maintained the previously established scoring criteria to enable comparability. Notably, we observed similar but generally less severe ALI features, particularly at 2 days post-infection (dpi), which largely resolved by 6 and 12 dpi. This temporal progression is consistent with the kinetics of lung injury and repair in the fLX model.

Regarding differences in scoring values compared to our prior Cell Reports publication, variations likely reflect differences in experimental conditions, the presence or absence of human immune components, and infection dynamics.

*2. The investigators see a minor population of chondrocytes present in their scRNAseq analysis. Typical lower airway or even lung parenchyma analyses usually do not pull this population of cells due to cartilage only being present in the trachea and upper airways. Is this population present due to the portions/locations of lung tissue that were collected or due to the fetal nature of the human lung tissue leveraged? Do the authors believe that initial fetal lung tissue collection (location) would impact and/or alter the findings seen? And is this at all reflected in the heterogeneity seen in the cell composition and responses?*

We thank this review of their comments. Residual amounts of cartilage are heterogeneously distributed across pieces of fetal lung tissues engrafted into animals, so it is expected to detect chondrocytes using scRNAseq. After having now run multiple infection studies using fLX-engrafted mice, leveraging multiple donors, we have not observed a significant impact of the tissue collection process, and from where fLXs derive in fetal lung tissues, on susceptibility to infection. However, in rare instances and in a donor-dependent fashion, fLX can display abnormal cartilage representation and are therefore excluded from our datasets.

*3. In figure 5 no club cells were noted. Is this not present in fLX or somehow lost in the analysis? Alterations in this compartment have been previously noted in earlier SARS-CoV-2 studies.*

We thank this reviewer for their comments. Club cells have been shown to be present in the lung xenografts of BLTL in the original publication (*Wahl et al., 2019. Nature Biotechnology*). We do identify club cells in our analysis, and they are classified into the airway secretory cluster (blue cells) as noted by their expression of *MUC5B*, *CYP2F1*, and *TSPAN8*. Further, we do not recover a high percentage of club cells (and non-immune cells) due to the use of Liberase TM digestion, which biases our recovery of immune cells over other cell types. We have now made mention of this in our method section titled “*Generation of single cell suspension from fLX”.* It is worth noting that the use of scRNAseq over single-cell nuclei also biases analysis toward hematopoietic cells.

*4. The authors identify a novel T-cell subcluster that when analyzed more carefully displayed low levels of CD4 and CD8 with high-level expression of macrophage markers which they dubbed double-negative T-cells. Given the unique nature of the model system and to ensure that this is not an artifact of the BLT flX model platform better linking of this population of cells to prior reports in human and in human viral infection would add greater strength and context to the manuscript.*

We thank this reviewer for bringing this point to our attention. We added literature references to our manuscript that describe the characterization of human monocyte-derived macrophage populations expressing CD3 (but not CD4/CD8) and how, in a mouse model, these populations increase at the site of infection following pleural inoculation with *Mycobacterium bovis (Rodriguez Cruz et al. Frontiers in Immuno. 2019).* We believe that placing our study in perspective with such work strengthens the potential significance of CD3-expressing macrophages in contributing to antiviral responses in the lung and lung-associated areas. Based on this additional literature review, we also decided to rename our myeloid-like double-negative T-cells (mDNT) population as CD3-expressing macrophage-like cells (MTC).

As our MTC subpopulation likely displays an experimental context-dependent phenotype through its unique blend of macrophages, lymphoid and mesenchymal markers, validating the existence of this specific subpopulation in humans poses however a considerable ethical and logistical challenge. Based on the existing literature about CD3-expressing macrophages in humans, we do believe that our work expands our understanding of how these cells respond to infection in humans and highlights the value of mouse models such as BLTL to understand their functions and phenotypic plasticity in human tissues.

*5. Figure 7K IF staining used to validate the enriched population seen by scRNAseq is hard to visualize due to the magnification. High mag images and report on the number of images this is representative would add strength to the data.*

We thank this reviewer for pointing this out. We have added Fig. 7K higher magnification images and clarified the number of images analyzed in the legends.

*6. The depletion of CD4 led to the persistence of SARS-CoV-2 infection which is a profound finding and the claim of chronic infection. Were later timepoints examined to demonstrate persistent infection? This is a really interesting model and no comment is made on the impacts of prolonged or “chronic infection” on the epithelial and mesenchymal/stromal compartments. As similar to that reported in prolonged human infections do the authors see the development of “pathological” fibroblasts and continued or dysregulated epithelial responses?*

We appreciate this reviewer’s comment. We agree that the BLTL model holds great promise for further investigating how chronic SARS-CoV-2 infection affects both the lung epithelial and mesenchymal compartments. The goal of this manuscript was to mainly focus on acute infection and identify immune signatures of infection resolution. For this reason, we limited our analysis to 12 dpi and did not investigate later timepoints. Studying the impact of prolonged/chronic infection on fLX in OKT4-treated mice at later time points (e.g., 30 dpi) will be the focus of future investigations, given that this represents an extension of this work rather than a critical piece of data that supports the current goal of our study. Focusing on pathological fibroblasts, (pre-)-fibrotic signatures, and epithelial dysregulations will be of primary interest, and so will be the potential genetic drifts of SARS-CoV-2. We have added a new section to our discussion related to this point in lines 639-657.

*7. While some limitations were noted with the BLT-L mouse model further comparison with prior humanized lung and immune system mice would provide greater contrast and comparison with the similarities and differences with such models and would help to better put the author’s work in context.*

We thank the reviewer for bringing this to our attention. The only human lung-immune system mouse models reported so far are the BLTL and HNFL mouse model, and we compare these two models together in lines 500-507. We have added another segment in our discussion about such comparisons in lines 664-668.

**Part II – Major Issues: Key Experiments Required for Acceptance**
Please use this section to detail the key new experiments or modifications of existing experiments that should be absolutely required to validate study conclusions.

*Generally, there should be no more than 3 such required experiments or major modifications for a "Major Revision" recommendation. If more than 3 experiments are necessary to validate the study conclusions, then you are encouraged to recommend "Reject".*

*Reviewer #1: The authors have previously established and characterized this model in their lab, which is already published. However, some key aspects should be revisited here in order to be able to interpret the data:*

*For example, NSG mice have been shown to be susceptible to infection of murine tissue by SARS-CoV-2. This could result in systemic effect that could (in part) influence responses in the lung xenografts. In fact, the authors also mention the presence of a minor population of murine cells in the scRNA-seq data, but excluded this from analyses. It would be important to specify what cell types were mostly associated, are these immune cells, or for example, endothelial cells involved in vascularization.*

Mice, including NSG mice, are not susceptible to SARS-CoV-2 infection because of an incompatibility between SARS-CoV-2 Spike and mouse ACE2. Transgenic expression, or humanization of the mouse ACE2 locus, can render mice susceptible to SARS-CoV-2 infection. In our study, NSG mice do not express human ACE2 and are therefore not susceptible to SARS-CoV-2 infection. Combined with the fact that SARS-CoV-2 is inoculated directly into fLX and does not disseminate through the blood (see Kenney et al 2022), this highlights that infection events defining fLX responses to infection (and hematopoietic infiltration) are likely strictly restricted to fLX.

However, this reviewer raises an interesting point about looking at the contribution of mouse cells in fLX in regulating fLX responses to infection. Therefore, we conducted an extended analysis of the mouse compartment. We identified the three clusters described in the new Figure S4 as i) Mesenchymal cells with a chondrocyte-like fibroblasts phenotype, ii) granulocytes, and iii) monocyte/macrophages. No endothelial cell subsets were detected (New Figure S4). Notably, monocyte/macrophages populations are enriched at 2 dpi, and a subpopulation of them is associated with viral RNA (New Fig. S4C,D) . Acknowledging the incompatibility between mouse ACE2 and SARS-CoV-2 WA-1 Spike, the presence of viral RNA is likely the result of mouse macrophage phagocytic activity, which is expected in such an infection context. The question of whether such mouse macrophage phagocytic activity contributes to infection resolution is partially addressed through our depletion experiment, which demonstrates that mouse monocyte/macrophages are not a major mediator of infection clearance in our experimental system. In this example, we show that human-specific depletion abrogates viral clearance, even though mouse macrophages/monocytes and granulocytes would not be impacted by depletion. Consistently with their minimal numbers compared to human cells, and the lack/limited cross-reactivity between many mouse cytokines/chemokines and human cytokines/chemokine receptors we do not expect that the mouse cells would have greatly impact clearance. We have expanded some segments of our result section to describe these mouse findings, and how our depletion experiments now also allow us to address a potential contribution of mouse cells in infection resolution mechanisms.

*The level of humanization (hCD45+) can vary between animals/donors etc. In Figure S3F, this ranges from 30%-100% in naïve animals. Was reconstitution determined prior to challenge, and if so, how did the authors deal with the variation with regard to grouping animals for the infection experiment?*

Peripheral engraftment is always measured by flow cytometry prior viral challenge. We have clarified this in the manuscript (Lines 740-742). We consider mice displaying a peripheral engraftment level of 40% to be acceptable for viral challenge, as indicated by the frequencies of CD45+ cells at 2 and 12 dpi in Figure S3F, left panel. However, a minor fraction of naïve mice (not challenged) displaying slightly lower engraftment levels (between 30-40%) were kept in our flow cytometry analysis in Figure S3F (Two mice out of 12). Overall, we have not observed any correlation between engraftment levels and susceptibility of fLX to infection, or in the ability of BLTL mice to clear infection. This is mainly due to the fact that while peripheral humanization represents one metric of successful engraftment, it does not necessarily correlate with fLX baseline human hematopoietic engraftment – a metric that is not accessible prior viral challenge and euthanasia. For this reason, we do not build mouse cohorts based on their peripheral engraftment levels. We adopt a randomized grouping as long as peripheral engraftment levels are acceptable (>40%). We have added this information in the method section of the Supplemental Information (Lines 54-57 of the Supplemental Information).

*Regarding controls, only naïve mice were included. From the M&M it seems naïve mice were not inoculated at all, a proper control would have been injection with PBS, give that injection of a relatively large volume of 50uL will have an effect on the structure of the tissue, and possibly the host response. In addition, particularly for the mesenchymal cell analyses, it would have been interesting to include data from lung-only animals. I believe the authors have this data available from their previous publication (Cell Rep. 2022 Apr 19;39(3):110714.).*

We thank this reviewer for requesting this clarification. All “naïve” mice in our studies were given PBS injections of the same volume. In the method section, the sentence “Mock/PBS infected mice were administered 50 μL of sterile 1X PBS” was added at lines 749-750. The lung-only animal data this reviewer is referring to is on a different mouse background (NRG not NSG) therefore direct comparison of the mesenchymal compartment (or other human compartment) could be challenging as the two different mouse models (NSG vs NRG) could respond differently to engraftment. Further, our previous study also does not include unfortunately scRNAseq analysis of fLX extracted from fLX-only mice. That being said, it would certainly be very interesting to perform a back-to-back comparison of the fLX environment using scRNAseq approaches upon human systemic hematopoietic engraftment or not to investigate the impact of such engraftment on fLX epithelial and mesenchymal signatures. However, we do believe that such an investigation falls beyond the scope of our current study.
 *From the text it is unclear what number of animals, donors and lungs tissues the results are based on. In M&M it says 3 donors were used, but not clear if these were used for each experiment, or whether in order to perform all experiments, a total of 3 donors were used. In the figure legends, it says the data are representative of 2 or 3 independent experiments. Is the data then represented as an average of the 2-3 experiments, or is data from 1 experiment used as representative for the 2-3 experiments? In the legend it also states that n=6-10 per time point, is this the number of animals, or the number of tissues? Is that than per experiment, or combined from 2-3 experiments. This is important because this could mean that data could be from 1 animal with 2 lung tissues per donor from 3 donors =6, or 3 animals from 1 donor.*

In this study and unlike our previous report (*Kenney et al. 2022. Cell Report*), each mouse was engrafted with a single fLX. No mouse had two fLX. Therefore, a data point represents a single fLX from a single mouse. We have further clarified the use of different donors across experiments in the method section (Lines 738-744) and in the Supplemental Information (Lines 51-52 and 57-60).

*Overall, the introduction is very long and could be reduced by summarizing from line 117-146, as this reads more as a discussion than introduction.*

We thank this reviewer for this comment. We have reduced our introduction accordingly.

*Reviewer #2: See above. No major experiments are required.*

*Reviewer #3: Kenney, et al. utilize a model of combined immune and fetal lung xenograft in mice to investigate the human tissue and immune responses to acute SARS-CoV-2 infection. While the model is interesting and may provide insights into factors that regulate acute SARS-CoV-2 infection, it relies largely on analysis of scRNA-Seq data. Strong conclusions are drawn from this scRNA-Seq data without functional validation, including conclusions about the role of endothelial cells, fibroblasts, mDNT cells, and CD4+ monocytes with little validation. Significantly more work should be done in one or more of these areas to validate claims made. Alternatively, specific validation of one of these conclusions with appropriate tempering of the conclusion of the role of these immune factors in regulating SARS-CoV-2 clearance throughout the manuscript would strengthen this report.*

We thank this reviewer for their comments. The major goal of our study was to capture the overall human signatures of acute SARS-CoV-2 infection resolution in human lung tissues, an endeavor that has not been accomplished yet. Our discussion then aims to place these signatures in perspective with the literature, speculate on overall clearance mechanisms, and illuminate multiple lines of new investigations that should focus on specific aspects of these signatures through entirely independent studies (such as MLC, iMO, PIM, endothelial regulation of infection clearance, mechanisms of chronic infection etc…. Our depletion experiments represent key evidence that our transcriptomic analysis can both guide and complement the functional dissection of infection resolution mechanisms in BLTL mice, which are amenable to such mechanistic studies. We have added statements in the discussion to further clarify the objective of our study (Lines 507-509 and 527-531). We have also ensured that all speculative statements are clearly identified as such.

*The authors claim that there is an overall reduction in T cells/ILCs, but infiltration of a “viral RNA-enriched T cell population displaying myeloid-like features in infected lung tissues.” If they’re not being grouped in with ILCs/T cells, are these MAIT cells or NK T cells?*

We appreciate this reviewer’s comment about the features of these cells. Unfortunately, identifying MAIT and NKT cells would require generating a new dataset that couples scRNAseq with scTCRseq. However, we do not think that the T/myeloid population (now referred to as MTC) 6 corresponds to MAIT cells and NKT cells for several reasons. First, innate-like T cells do not have a specific transcriptional signature when compared to conventional tissue-resident T cells from the same tissue, and are thus not expected to cluster separately. Furthermore, the characteristics of MTC (cluster 6 among T-cell clusters in Figure 6) do not fit with the general features of innate-like or memory T cells, namely they do not highly express transcription factors associated with innate-like/memory T cells (e.g., *ZBTB16, ID2, Id3*), nor transcription factors associated with the primed effector status of such cells (e.g., *RORC, TBX21, EOMES*) (See below). Therefore, we do not believe that these are MAIT or NK T cells.

*Some amount of validation should be done to determine what these cells are. Is it possible this signature is a result of a technical issue with cell collection (doublets) or analysis. Is it possible myeloid cells are taking up other infected cells leading to this signature?*

We appreciate this reviewer raising these possibilities. Although we have used standard doublet discriminator approaches, these approaches are imperfect, and some doublets could indeed have been included in our analysis. However, several pieces of evidence suggest that these cells (T-cell cluster 6) are unlikely to be doublets or myeloid cells that are taking up infected cells. This is particularly notable as the number of genes expressed by these cells is limited, whereas doublets would express more genes than single cells (see below cluster #6; as compared to the other T-cell clusters described in Figure 6). Further characterization of the phenotype of these cells will be the focus of a future study.

A potential explanation for the unique transcriptional profile of MTC is the loss of cellular identity/dedifferentiation due to a high enrichment in SARS-CoV-2 RNA. Indeed, our scRNA-seq data reveals that MTC (Cluster 6 below) show decreased expression of T cell transcription factors (e.g., *TCF7*, *BCL11B*, *GATA3*), as well as the upregulation of transcription factors expressed in alternative lineages, and not only myeloid lineages (*SOX4, TCF4, EGR1,* and *CEBPD*) (see below).

*Are these cells productively infected?*

We thank the reviewer for bringing up this point. It would be very difficult to conclude if the T cells in cluster 6 are productively infected because we cannot assess if they are producing viral particles. However, several scRNAseq-associated signatures suggest active viral RNA replication/translation, and some of these elements have been added to our manuscript (new Figure S6). For instance, endogenous mRNA expression is shut down (see cluster 6, left panel below), but ribosomal transcripts were overrepresented, suggesting high translational activity despite the low levels of mRNA (see cluster 6, right panel below). This indicates that the endogenous translational machinery is hijacked to produce viral components (*Banerjee AK et al., 2020; Finkel Y et al., 2021*).

The biological process most enriched in transcripts downregulated in cells from cluster 6 (our population of interest) compared to cells from other T-cell clusters relates to mRNA production (see bar chart below), including many transcripts encoding for the basal mRNA transcriptional machinery (e.g., components of RNA pol II:*POLR2A, POLR2J3.1*, and preinitiation complexes: *TAF1D, TAF7, CCNH, EIF4A1*; See violin plots below).

Additionally, ZC3HAV1 was low and is a known antiviral protein that inhibits viral replication of SARS-CoV-2 and other viruses by recruiting cellular RNA degradation machineries to degrade viral RNAs (*Lee S et al. Mol Cell 2021; Zimmer MM et al Nat Com, 2021*).

Although not specific to infection, our T/myeloid (cluster 6) population displayed several other features compatible with productive infection. Cluster 6 myeloid/T cells have key features of productively infected cells, including:

1) Elevated mitochondrial RNA encoded transcripts (*Miller et al 2021, Sci Rep; Shin et al 2024, Sig Trans and Targ Therapy*; See Fig.S5A)

2) Cellular responses to stress; see notably SOD2, a mitochondrial detoxification which destroys superoxide anion radicals

3) cellular macromolecule biosynthetic process (other than ribosome):

4) non-membrane-bounded organelle assembly:

5) Response to elevated platelet cytosolic Ca2+ :

*Further, are T cells actually leaving the tissue as the authors suggest or is the change in frequency as shown by scRNA-Seq due to infiltration of other cell subsets? In this model where virus is rapidly cleared from tissue explants after day 2 p.i., are T cells required? Is appropriate T cell priming occurring in lymph nodes and is the explanted tissue sufficiently vascularized for infiltration of these cells?*

We thank this reviewer for their comment. We have clarified the text to indicate that decreased T-cell frequencies could be either due to T-cell loss in fLX and/or increased in myeloid extravasation. However, mIHC in Figure 7K does suggest a significant reduction in CD3+ cells upon infection. Our depletion experiment suggests that T-cells are not major drivers of infection resolution, at least through antigen-specific mechanisms, as this would not be compatible with the rapid clearance timeline (less than six days post-infection) and the fact that NSG mice (similarly to many other humanized mouse models) have atrophied lymph nodes. Therefore, any potential contribution of T-cells in the infection resolution process, which is reflected by evidence that OKT3 depletion fails to abrogate infection resolution in 50% of the fLX, is likely cytokine/chemokine-mediated. Our previous report (*Kenney et al. 2022. Cell Report*) has reported effective fLX vascularization through myeloid extravasation and evidence that intravenously delivered Nanoluc substrate enable to detect replication of a NanoLuc-expressing SARS-CoV-2 virus in fLX. The major monocytic infiltration observed in our study at 2 and 12dpi also support sufficient vascularization; and so are more recent studies as well (*Olivier Escaffre et al. 2024. JVI*)

*The authors claim a critical role for patrolling monocytes (PIM) in regulating acute infection. These cells should be looked at to determine whether they are actually patrolling in the vasculature and provide validation of these subsets within the tissue beyond scRNA-Seq.*

We thank this reviewer for their comment. We agree that it would be interesting to investigate further the role of PIM. However, we feel that such an investigation would represent, and likely benefit from, an entirely independent study and framework. The goal of our current study is to provide a broad overview of the different immune signatures associated with SAR-CoV-2 infection resolution and to highlight multiple avenues for future independent investigations, each focusing on mechanistically exploring a specific aspect of these signatures.

*The conclusion but this is based on assessment of scRNA-Seq and broad depletion of CD4 compared to CD3. Based on depletion studies, the authors claim T cells are not required for protection. However, it seems T cells play a role based on 50% of anti-CD3 treated tissues still containing virus on day 12 p.i. compared to 73% with anti-CD4 treatment. More specific assessment of these CD4+ monocyte populations and more specific depletion strategies should be used to justify this claim given the majority of the “persistence” of virus at day 12 p.i. is attributed to anti-CD3 treatment.*

We thank this reviewer for their comment. We do not claim that T-cells are not required for infection resolution, and we apologize for any misunderstanding. Instead, we argue that our results support that CD4+ cells play a more prominent part than CD3+ cells in resolving infection, aligning with our scRNAseq data, consistent with the fact that i) more fLX cannot resolve infection upon OKT4 treatment, and that ii) among fLX in which infection resolution is abrogated, OKT4-treated animals displayed a lung viral titer more than 21-fold higher (mean Log10 PFU/mg tissue = 3.264) compared to OKT3-treated animals (mean Log10 PFU/mg tissue =1.930). However, we do agree that we cannot exclude a contribution of CD3+ cells at this stage (whether its CD3-expressing macrophages-like cells or T-cells), albeit such a contribution remains unclear. A segment of our discussion in our original manuscript was dedicated to discussing what such a contribution could entail. Unfortunately, no other human monocyte-specific depletion strategy has been validated in vivo to enable the conduct of confirmatory deletion experiments; which is why we extensively leveraged mIHC for orthogonal validations in our study. We do not understand what this reviewer means by “the majority of the “persistence” of virus at day 12 p.i. is attributed to anti-CD3 treatment.” Does the term persistence here relate to viral titers and/or frequencies of fLX with detectable viral titers? In either case, our data do support that anti-CD4, and not anti-CD3 treatment, yields both to a higher frequency of fLX with persisting viral titers, and higher viral titers in fLX defective for infection clearance.

*Does treatment with OKT3 or OKT4 lead to clearance in all infected explant lungs?*

These data are presented in Figure 9B. OKT4 treatment abrogated infection clearance in 72% of animals, while OKT3 treatment abrogated infection clearance in 50% of the animals. Mean fLX viral titer in OKT4-treated mice was significantly different from that of isotype-treated mice, while it was not the case for OKT3-treated mice. Furthermore, as shown in Fig. S6B, among fLX in which infection resolution is abrogated, OKT4-treated animals displayed a lung viral titer more than 21-fold higher (mean Log10 PFU/mg tissue = 3.264) compared to OKT3-treated animals (mean Log10 PFU/mg tissue = 1.930). Together, these findings support a more significant contribution of CD4+ cells over CD3+ cells in driving infection resolution.

*Additionally, only day 12 is assessed. Are there earlier changes in virus titers or delays in clearance in all antibody depletion treatments?*

Due to limitations in the number of mice available for this study, only one time point could be assessed for our depletion experiments. As infected, but non-treated BLTL mice have all cleared the infection by day 12, we considered this the best time point (vs. day 6) to evaluate evidence of abrogated viral clearance mechanisms.

*In the last figure and at the end of the results and in the discussion, the authors show and discuss “ExiMO” but this subset is not defined in any manner. This should be clarified. In general whether monocytes or specific subsets of monocytes are important in mediating protection should be addressed more specifically through scientific data and discussion of the existing literature.*

We thank this reviewer for bringing this to our attention. This was a mistake on our end. ExiMO should be say iMO. We have made the appropriate corrections.

**Part III – Minor Issues: Editorial and Data Presentation Modifications**
Please use this section for editorial suggestions as well as relatively minor modifications of existing data that would enhance clarity.

*Reviewer #1: Line 159. Please provide a rationale for using the 2019-nCoV/USA_WA1/2020 strain, as this has not been circulating for several years.*

This project was initiated in late 2020 with the WA-1 strain and finalized in 2023; we decided to maintain consistency with the strain throughout the project.

*Line 196. The authors should also test for non-neutralizing antibodies against S or N, for example, which are typically present in higher concentrations and may still play a role in clearance.*

Unfortunately, we did not have any sera available for these experiments.

*Line 204. While interesting, a link with adaptation seem an overreach. The authors should also sequence virus from murine tissue if indeed this is a matter of adaptation.*

We have toned down the language in this section. No mouse cells or tissues can be productively infected in this mouse model due to the lack of compatibility of SARS-CoV-2 Spike with mouse ACE2 (see our response above).

*Line 250. These data suggest the potential infection of T cells. Is there a way to confirm whether active replication is taking place in these cells? Could you test for sgRNA in PBMC for example?*

We have added new information in the manuscript that could support productive infection of these cells (See our answers to reviewer #3 above). For definitive evidence, the specific T/myeloid subpopulation will need to be sorted out of fLX at 2 dpi, and the small numbers of these cells may make this challenging. This could be the focus of follow up studies.

*Line 393. Is there evidence of viral RNA in endothelial cells?*

We have not detected viral RNA in endothelial cells (Fig. 4A).

*Line 688. Please specify at what week post implantation, the reconstitution was assessed.*

We have added this information in the manuscript (Lines 741; Line 54 of the Supplemental Information).

*Reviewer #2: See above. No major experiments are required.*

*Reviewer #3: Critical details regarding the model are not included. The supplemental methods section contains the statement: “Human immune reconstitution was determined by flow cytometry at weeks post implantation.” However, the timepoint at which reconstitution by assessment of blood is not determined. Additionally, how many weeks post implantation infection is occurring at is not included in the methods.*

We have added this information in the manuscript (Human immune reconstitution : Line 741 and line 54 of the Supplemental Information; Time of challenge: Lines 746-747).

*In several instances conclusions are made from single histological images without any type of quantification 9. These include: quantification of SARS-CoV-2 IHC staining across multiple tissues in Figure 1 E-L, Figure 2 C-D describing features of damage with no broader naïve controls, quantification of SARS-CoV-2 IHC staining across multiple tissues in Figure 9C (in this case OKT3 image shows no viral antigen with Figure 9C shows half the animals still have viral antigen), and quantification of MHC-I IHC staining across multiple tissues in Figure 10.*

We thank this reviewer for their suggestions. For Figure 1 E-L, our framework for this study involves leveraging PFU for viral quantification while utilizing IHC for qualitative assessment of antigen distribution. For Figure 2 C-D, control can be seen in panel A. For Figure 9C, we have added IHC images from OKT3-treated mice that do not exhibit viral clearance, as well as IHC images from OKT4-treated mice showing varying levels of viral infection. For Figure 10, images are representative of two fLX for each experimental condition. We have clarified this in the legend of Figure 10.
